# IDESS: a toolbox for identification and automated design of stochastic gene circuits

**DOI:** 10.1093/bioinformatics/btad682

**Published:** 2023-11-21

**Authors:** Carlos Sequeiros, Manuel Pájaro, Carlos Vázquez, Julio R Banga, Irene Otero-Muras

**Affiliations:** Computational Biology Lab, MBG-CSIC (Spanish National Research Council), 36143 Pontevedra, Spain; Department of Mathematics, University of Vigo, Escola Superior de Enxeñaría Informática, Campus Ourense, 32004 Ourense, Spain; Department of Mathematics and CITIC, Universidade da Coruña, Campus Elviña s/n, 15071 A Coruña, Spain; Computational Biology Lab, MBG-CSIC (Spanish National Research Council), 36143 Pontevedra, Spain; Computational Synthetic Biology Group, Institute for Integrative Systems Biology (I2SysBio), CSIC-UV, 46980 Paterna, València, Spain

## Abstract

**Motivation:**

One of the main causes hampering predictability during the model identification and automated design of gene circuits in synthetic biology is the effect of molecular noise. Stochasticity may significantly impact the dynamics and function of gene circuits, specially in bacteria and yeast due to low mRNA copy numbers. Standard stochastic simulation methods are too computationally costly in realistic scenarios to be applied to optimization-based design or parameter estimation.

**Results:**

In this work, we present IDESS (Identification and automated Design of Stochastic gene circuitS), a software toolbox for optimization-based design and model identification of gene regulatory circuits in the stochastic regime. This software incorporates an efficient approximation of the Chemical Master Equation as well as a stochastic simulation algorithm—both with GPU and CPU implementations—combined with global optimization algorithms capable of solving Mixed Integer Nonlinear Programming problems. The toolbox efficiently addresses two types of problems relevant in systems and synthetic biology: the automated design of stochastic synthetic gene circuits, and the parameter estimation for model identification of stochastic gene regulatory networks.

**Availability and implementation:**

IDESS runs under the MATLAB environment and it is available under GPLv3 license at https://doi.org/10.5281/zenodo.7788692.

## 1 Introduction

The field of Synthetic Biology is making rapid progress toward achieving fully automated design of DNA sequences to reprogram cells with novel functions and capabilities. Software tools for the automated design of biocircuits can be categorized based on whether they focus on the steady-state input–output behavior (Nielsen *et al.* 2016) or address the dynamics of the biocircuit (Otero-Muras *et al.* 2016, Tanevski *et al.* 2016, Sents *et al.* 2023), considering the underlying mathematical models. One milestone within the first category is CELLO—see the most recent version by Jones *et al.* (2022)—based on Boolean logic gates. Importantly, CELLO is the first design environment that has been calibrated with experimental data (originally in *E.coli*, and most recently also in yeast *S.cerevisiae*), and it outputs the DNA sequence required to implement the logic circuit provided as an input. Within the second category, SYNBADm (Otero-Muras *et al.* 2016) tackles high levels of biological complexity by combining dynamic models based on Ordinary Differential Equations (ODEs) with multiobjective optimization across parameter and topology spaces. The tool takes as input the design target behavior defined by the user and delivers as output the specific biocircuit (topology and parameters) needed to achieve this desired behavior. The scope of SYNBADm is restricted to the deterministic regime (scenarios in which the effects of molecular noise can be neglected).

It has been extensively reported how noise can play a crucial role in gene circuit engineering (Beal 2017). There are different sources of noise (Pischel *et al.* 2017) that can affect the dynamics of biocircuits, including the inherent stochasticity of the biochemical reactions involved (intrinsic noise). The impact of stochasticity on the dynamics of gene circuits when the copy numbers are low is well established. However, automated design of biocircuits under the effects of molecular noise is challenging due to the computational cost of stochastic simulations using standard methods.

In this work, we present Identification and automated Design of Stochastic gene circuitS (IDESS), a software toolbox for automated design and identification of biocircuits in the stochastic regime. IDESS is capable of simulating stochastic biocircuits very efficiently using GPU acceleration for simulation and global optimization. It includes CPU and GPU parallel implementations of the Stochastic Simulation Algorithm (SSA) (Gillespie 1976) and the semi-Lagrangian Simulation method in SELANSI (Pájaro *et al.* 2018). This semi-Lagrangian numerical method simulates a Partial Integro-Differential Equation (PIDE) model describing the biocircuit dynamics. One significant advantage of this method is its efficiency to compute the whole probability distribution of the random variables (protein levels) describing the state of the system over time. IDESS utilizes Global Optimization solvers capable of optimizing efficiently over high dimensional search spaces of continuous real and discrete integer variables, including Mixed Integer Nonlinear Programming (MINLP) solvers to optimize simultaneously across parameter and topology search spaces.

## 2 Main features

IDESS performs simulation, automated design and parameter identification of gene regulatory circuits combining efficient methods for simulation of stochastic gene regulatory networks with global optimization. The toolbox is implemented in MATLAB under WINDOWS environment. The main functionalities of the toolbox are summarized next.

- **Simulation of stochastic gene regulatory circuits:** IDESS implements the SSA (Gillespie 1976) and the semi-Lagrangian method (Pájaro *et al.* 2018) that solves the PIDE approximating the Chemical Master Equation (CME) of gene regulatory networks. Both methods can take advantage of GPU-parallelization in order to improve performance.
- **Model calibration of stochastic gene regulatory networks:** IDESS performs maximum likelihood estimation to estimate the parameters of a gene regulatory network model that best fit the observed/experimental data. The likelihood is optimized across the parameter space using enhanced Scatter Search as implemented in the MEIGO software suite for global optimization (Egea *et al.* 2014).
- **Automated design of synthetic gene circuits:** IDESS optimizes a performance function encoding the target behavior of the circuit. The optimal design problem is solved as a MINLP problem (where network topology and parameters are optimized simultaneously) using MEIGO (Egea *et al.* 2014).

**Figure 1.**
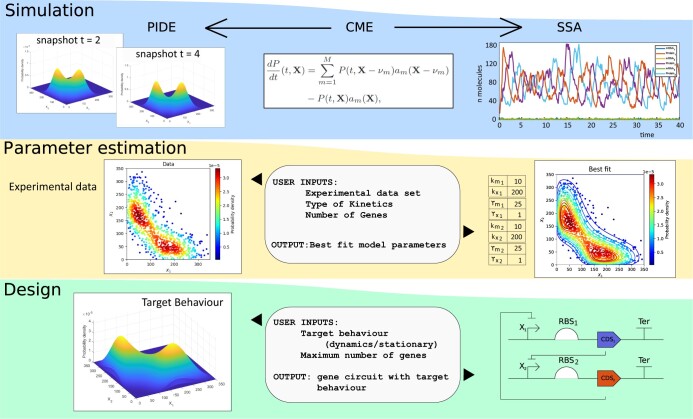
Main features of IDESS: (i) simulation of biocircuit dynamics by Semi-Lagrangian (PIDE model) or SSA Algorithms, (ii) parameter estimation from experimental data for model identification, and (iii) automated design of biocircuits, delivering synthetic gene circuits (topology and parameters) with predefined target behaviors. IDESS applies CPU and GPU parallel implementations of stochastic simulation and global optimization to accelerate computing.

In Fig. 1, we present a sketch of the main features of the toolbox. In the following section, we provide additional details regarding the implementation of these functionalities.

## 3 Implementation

### 3.1 Simulation

A gene regulatory network or biocircuit formally consists of a set of *N* genes that are transcribed into mRNAs and then translated into proteins, which in turn regulate the expression of genes in the network (see Table 1). The CME that describes the dynamics of stochastic gene regulatory circuits consists of the following system of ODEs (see Ge and Qian 2013):

(1) $$\frac{dP}{dt}(t,X)=\sum_{m=1}^{M}P(t,X-\nu_{m})a_{m}(X-\nu_{m})-P(t,X)a_{m}(X),$$

where *P* denotes the probability distribution associated to the *N* proteins, $\nu_{m}$ captures the stoichiometry and $a_{m}$ the propensity of each of the reactions and *M* is the number of reactions. The dimension of this ODE system exploits in realistic scenarios making unfeasible or impractical to solve it directly. The PIDE model that we use to approximate the CME is formulated as follows (see Pájaro *et al.* 2017):

(2) $$\begin{array}{c} \frac{\partial P}{\partial t}(t,X)=\sum_{i=1}^{N}\frac{\partial }{\partial X_{i}}\left[\gamma_{X}^{i}(X)X_{i}P(t,X)\right]\\ +\sum_{i=1}^{N}\left(k_{m}^{i}\int_{0}^{X_{i}}\beta_{i}(X_{i}-Y_{i})c_{i}(Y)P(t,Y)dY_{i}\right). \end{array}$$

**Table 1.** Reactions and propensities of a gene regulatory circuit with *n* genes and *m* reactions.^a^

Reaction	Propensity
$\emptyset \to \mathrm{mRN}A_{1}$	$k_{m}^{1}\cdot c_{1}(X)$
$\mathrm{mRN}A_{1}\to \mathrm{mRN}A_{1}+X_{1}$	$k_{x}^{1}\cdot \mathrm{mRN}A_{1}$
$\mathrm{mRN}A_{1}\to \emptyset$	$\gamma_{m}^{1}\cdot \mathrm{mRN}A_{1}$
$X_{1}\to \emptyset$	$\gamma_{x}^{1}\cdot X_{1}$
⋮	⋮
$\emptyset \to \mathrm{mRN}A_{n}$	$k_{m}^{n}\cdot c_{n}(X)$
$\mathrm{mRN}A_{n}\to \mathrm{mRN}A_{n}+X_{n}$	$k_{x}^{n}\cdot \mathrm{mRN}A_{n}$
$\mathrm{mRN}A_{n}\to \emptyset$	$\gamma_{m}^{n}\cdot \mathrm{mRN}A_{n}$
$X_{n}\to \emptyset$	$\gamma_{x}^{n}\cdot X_{n}$

a $k_{m}$ and $k_{X}$ are the transcription and translation rate constants and $\gamma_{m}$ and $\gamma_{X}$ are the degradation rate constants of mRNA and protein, respectively. $c_{m}(X)$ is the input function encoding the regulation by proteins.

IDESS implements both the SSA algorithm, and the semi-Lagrangian method solving the PIDE model. It is important to note that, while the SSA algorithm provides realizations of the dynamics (time course trajectories), the PIDE model provides the whole probability distribution over time. Moreover, IDESS includes the optional use of GPU parallelization to run simultaneously a large number of SSA simulations or to greatly speed up the semi-Lagrangian algorithm for solving the PIDE model. It is also worth noting that, although the original formulation takes into account only intrinsic noise, extrinsic noise can be easily incorporated through the input function $c_{i}(Y)$ in Equation 2.

### 3.2 Model calibration

In a model calibration problem we start from a regulatory network with fixed topology, and estimate the parameters that maximize the probability of reproducing a given set of data. This probability is provided by the likelihood function. IDESS can perform model calibration from time course data, histogram time series or multidimensional displays (dot displays or contour maps from flow cytometry analyses, e.g.). The parameters to be estimated include the unknown transcription, translation and degradation rate constants as well as cooperativities of the regulation. Parameters with known values are fixed. At each iteration of the optimization algorithm, the candidate vector of parameters is supplied to the simulation algorithm and the cost function is evaluated. The Kullback–Leibler divergence measures the distance between probability distributions (Sequeiros *et al.* 2023b). IDESS includes as an illustrative example the parameter estimation of a Toggle Switch model from time series of protein level distributions displayed in 2D plots (see Fig. 1).

### 3.3 Automated design

The automated design problem consists of finding the circuit topology and parameters that lead to a target functionality defined *a priori*. The design objective is encoded in a cost function that can take different forms depending on the desired behavior. Design objectives might include: (i) target dynamics (evolution of the probability distribution of the proteins over time), (ii) target stationary distribution, (iii) bimodal switches with given distance between modes or fixed probabilities of certain domains, (iv) capacity of adaptation upon external signals, and (v) oscillatory behavior with given metrics or optimal robustness against noise.

The decision variables for the design include integer variables (topology) and real parameters (transcription, translation, degradation rate constants, cooperativities of the regulation, and promoter leakages). The user can fix the values of some of those parameters (if applicable to the specific design problem).

IDESS includes as an illustrative example the design of a three-gene circuit with oscillatory behavior. In this case, the objective function is the second peak of the autocorrelation function of the protein stochastic dynamics, such that the biocircuit obtained as a result of the optimization has maximum robustness against molecular noise (Sequeiros *et al.* 2023a).

## Data Availability

Data and code underlying this article are available at: https://doi.org/10.5281/zenodo.7788692.
